# Differential in vitro cytotoxic effects and metabolomic insights into raw and powdered Manuka honey through UPLC-Q-TOF-MS

**DOI:** 10.1038/s41598-024-68387-7

**Published:** 2024-07-30

**Authors:** Ienas Idriss, Abdelmoneim H. Ali, Aftab Alam, Maria Fernandez-Cabezudo, Mutamed Ayyash, Basel K. al-Ramadi

**Affiliations:** 1 Department of Medical Microbiology and Immunology, College of Medicine and Health Sciences, United Arab Emirates University (UAEU), Al Ain, UAE; 2 Department of Food Science, Faculty of Agriculture, Zagazig University, Zagazig, 44511 Egypt; 3 Department of Biochemistry & Molecular Biology, College of Medicine and Health Sciences, United Arab Emirates University (UAEU), Al Ain, UAE; 4 Department of Food Science, College of Agriculture and Veterinary Medicine, United Arab Emirates University (UAEU), P.O. Box 15551, Al Ain, UAE; 5 Zayed Center for Health Sciences, United Arab Emirates University (UAEU), Al Ain, UAE; 6 ASPIRE Precision Medicine Research Institute Abu Dhabi, United Arab Emirates University, Al Ain, UAE

**Keywords:** Manuka honey, Cancer, Anti-proliferative effect, Untargeted metabolomics analysis, UPLC-Q-TOF-MS, Metabolomics, Carbohydrates, Cancer

## Abstract

Manuka honey (MH) has garnered much attention due to its remarkable antimicrobial, anticancer, immunomodulatory and wound-healing properties. This study compared the antiproliferative effects of raw and powdered MH (pMH) on various human and murine cancer cell lines. A detailed metabolomics analysis was also carried out using untargeted ultrahigh-performance liquid chromatography-quadrupole-time-of-flight-mass spectrometry (UPLC-Q-TOF-MS) to compare the constituents in raw MH and pMH. The results of the viability studies showed that both raw MH and pMH caused a dose-dependent inhibition of tumor cell growth at concentrations of > 1% w/v (equivalent to ~ 10 mg/ml). A differential susceptibility to MH was observed among the cell lines with the human MDA-MB-231 and A549 cells and murine B16.F10 cells being relatively resistant to MH while the murine MC38 colorectal adeno-carcinoma cells showing the most sensitivity. The effect of raw MH and pMH on cell viability was validated using 2 indepndent assays. Metabolomics analysis detected 2440 compounds, out of which 833 were successfully identified. Among these, 90 phytochemical compounds, predominantly comprising terpenoids, flavonoids, coumarins and derivatives, and phenylpropanoic acids, and 79 lipids were identifiable. Significant differences in 5 metabolite classes, including flavonoids, phenols, terpenoids, carbohydrates, and organic acids were observed between the raw and pMH. Moreover, several altered metabolic pathways were identified in pMH compared to raw MH, such as energy metabolism, amino acid metabolism, and various other pathways that collectively influence biological functions associated with cellular growth, signaling, and stress response.

## Introduction

Honey, a flavorful natural sweetener, is produced by bees from flower nectar and plant secretions. Comprising over 200 compounds, honey is a complex substance with approximately 60–75% carbohydrates, including monosaccharides, disaccharides, and polysaccharides. Additionally, it contains free amino acids and proteins (2%), minerals (1%), acids (1%), enzymes and vitamins (1%), carotenoid-like substances, polyphenols, and Maillard reaction products^1^. The bioactivity of honey is primarily attributed to its bioactive molecules, with polyphenols, in particular, playing a significant role^2,3^. Polyphenols, natural phytochemical products of plant secondary metabolism, are crucial for the antioxidant and anticancer activities of honey^4,5^. Manuka honey (MH), produced by Apis mellifera honeybees from New Zealand Manuka trees (*Leptospermum scoparium*), is a distinctive floral variety^6^. The global MH market, valued at $455.4 million in 2021, is projected to reach $776.4 million by 2031, growing at a compound annual growth rate of 5.5% from 2022 to 2031. Owing to its active biomolecules, MH has demonstrated effective antioxidant, anticancer, and immunomodulatory properties^7–9^.

Due to its complex composition, untargeted metabolomics serves as an inclusive and unbiased methodology to assess antioxidant activities in honey^10^. The primary goal of untargeted metabolomics is to identify various metabolites, defining trends or characteristics without the need for compound quantification^11^. Employing spectroscopy or chromatography techniques, the metabolite profile in the analyzed sample is assessed. Chemometric analysis, a modern chemical analysis approach, is then applied to simplify complex results, derive valuable information, and enhance analysis comprehensibility^12^. Metabolomics methodology, coupled with untargeted chemometric analysis, has gained prominence in identifying bioactive compounds or chemical markers in various food matrices, including honey^13,14^.

Ultra-performance liquid chromatography coupled with quadrupole-time of flight-mass spectrometry (UPLC-Q-TOF-MS) and gas chromatography-mass spectrometry (GC–MS) have been previously employed to identify potential antioxidant activity-based metabolites in honey^15^. GC–MS, preferred for its high sensitivity, robustness, and ability to provide a comprehensive metabolite range within a single run, is complemented by UPLC-Q-TOF-MS. The latter, known for its soft ionization, high quantity, and good metabolite coverage, is particularly adept at revealing low-abundant and high molecular weight metabolites in targeted samples^16^. Given that no single technique can measure all metabolites in food and biological samples, the combined use of UPLC-Q-TOF-MS and GC–MS provides a comprehensive chemical analysis of honey. Other analytical techniques, such as nuclear magnetic resonance (NMR) spectroscopy and Fourier transform infrared (FTIR) spectrometry coupled with attenuated total reflectance (ATR), have also been utilized in untargeted metabolomics analysis to assess variations in metabolite levels^17,18^.

Recently, dried MH products have become available in the market. In their powdered form, and given their solubility and ease of preparation, they represent an attractive alternative means by which to administer honey orally or systemically. The main objective of this study was to perform untargeted metabolomics analysis of raw MH and pMH using ultrahigh-performance liquid chromatography-quadrupole-time-of-flight-mass spectrometry (UHPLC-Q-TOF–MS) to determine their characteristics and uncover any potential differential metabolomics content. In addition, given the previously described anti-cancer properties of MH^19–21^, we determined the cytotoxic capacity of the two types of MH against various human and murine cancer cell lines in our effort to compare their functional activities.

## Materials & methods

Raw Manuka honey (MH; UMF® 20+) was acquired from ApiHealth (Auckland, New Zealand). Powdered Manuka honey (pMH), generously provided by GS Foods Ltd (Marlborough, New Zealand), comprises 70% MH solids and 30% maltodextrin. All reagents were freshly prepared under aseptic conditions in sterile culture medium and passed through a 0.2 µM filter (Corning®, Germany) before addition to the cells in culture.

### Cancer cell lines

The human breast cancer cell line (MDA-MB-231) was supplied by Dr. Salem Chouaib (Institut Gustave Roussy, Villejuif, France), and the human lung cancer cell line (A549) was a gift from Dr. Samir Attoub (College of Medicine and Health Sciences, UAEU). The cell lines were maintained in either complete Dulbecco's Modified Eagle Medium (DMEM) for MDA-MB-231 cells or Roswell Park Memorial Institute medium (RPMI) for A549 cells, supplemented with 10% fetal calf serum (FCS) and antibiotics (Gibco-ThermoFisher Scientific, Waltham, MA, USA), as previously described^19^. The murine colon adenocarcinoma MC38 cell line was kindly provided by Dr. Jo Van Ginderachter (Vrije University Brussel, Belgium). The mouse colon carcinoma cell line CT26 was a gift from Dr. Siegfried Weiss (Helmholtz Centre for Infection Research, Braunschweig, Germany). The murine B16.F10 melanoma cancer cell line was a gift from Dr. Robert Zeiser (University Medical Center, Freiberg, Germany). Murine cell lines were also maintained in DMEM supplemented with 10% FCS plus antibiotics^21,22^. Cells were incubated at 37°C in a humidified atmosphere of 5% carbon dioxide and used in the log phase of growth. In all experiments, cell viability exceeded 95% at the start of in vitro culture.

### Cell proliferation assay

Cell viability was assessed using two separate assays, namely the CellTiter-Glo® Luminescent Cell Viability Assay (Promega, Madison, WI, USA) and the BrdU cell proliferation Elisa kit (Abcam). the CellTiter-Glo® assay measures viability by quantifying ATP levels in cell culture wells and was carried our as previously described^20^. The BrdU assay detects BrdU incorporation into newly synthesized DNA in actively proliferating cells. Both assays were conducted following the manufacturer’s instructions, and luminescence and absorbance were recorded at specified time points using a TECAN Infinite 200 microplate reader. Tumor cells (5 × 10^3^ cells/well) were cultured with different concentrations of MH and pMH1 (range 0.1–2.5%; w/v, equivalent to 1–25 mg/ml) in 96-well plates for 24 h or 48 h. As positive controls, cells were incubated in culture medium without any additions. All determinations were done in duplicates or triplicates or as otherwise indicated. Readings were normalized after subtracting background signal (wells containing medium alone; negative controls). Data are presented as a percentage of cell viability compared to untreated cells (positive controls).

### Untargeted metabolomics analysis

Triplicate samples of raw MH or pMH were subjected to metabolomics analysis using the UPLC-MS/MS technique. Serum samples stored at − 20 °C were kept at 4 °C until thawing. Then, each sample (100 μL) was mixed with 700 μL of extractant containing internal standard 1 (methanol/acetonitrile/water; 4:2:1), mixed for 1 min, kept at − 20°C refrigerator for 2 h, followed by centrifugation for 15 min at 25,000*g* and 4 °C. After centrifugation, 600 μL of the supernatant was transferred to a split new stainless steel electro-polished tube. The solvent was evaporated, and 180 μL of methanol/pure water (1:1) was added and mixed for 10 min until all dissolved in the reconstituted solution. The samples were centrifuged for 15 min at 25,000*g* and 4 °C, and the supernatant was transferred to a new tube. Then, 20 μL of each sample was used for UPLC-MS/TS analysis.

### UPLC-MS analysis and data processing

Analysis was carried out using Waters UPLC I-Class Plus (Waters, USA) Tandem Q Exactive high-resolution MS (Thermo Fisher Scientific, USA) for separation and detection of metabolites. Chromatographic separation was performed on a Waters ACQUITY UPLC BEH C18 column (1.7 μm, 2.1 mm × 100 mm, Waters, USA), and the column temperature was maintained at 45 °C. The mobile phase consisted of 0.1% formic acid as mobile phase A and acetonitrile as mobile phase B in the positive ion mode. In the negative ion mode, the first mobile phase consisted of 10 mM ammonium formate, and acetonitrile was the second mobile phase. The gradient conditions were as follows: 0–1 min, 2% B; 1–9 min, 2–98% B; 9–12 min, 98% B; 12–12.1 min, 98% B to 2% B; and 12.1–15 min, 2% B. The flow rate was set at 0.35 mL/min, and the injection volume was 5 μL.

Q Exactive (Thermo Fisher Scientific, USA) was applied to perform primary and secondary MS data acquisition. The full scan range was 70–1050 m/z with a resolution of 70,000, and the automatic gain control (AGC) target for MS acquisitions was set to 3 × 106 with a maximum ion injection time of 100 ms. Top three precursors were selected for subsequent MS/MS fragmentation with a maximum ion injection time of 50 ms and resolution of 17,500; the AGC was 1 × 105. The stepped normalized collision energy was set to 20, 40, and 60 eV. Electrospray ionization parameters were set as follows: sheath gas flow rate of 40 mL/min, auxiliary gas flow rate of 10 mL/min, positive-ion mode spray voltage of 3.80 kV, negative-ion mode spray voltage of 3.20 kV, capillary temperature of 320 °C, and auxiliary gas heater temperature of 350 °C.

### Metabolite ion peak extraction and metabolite identification

After importing the offline mass spectrometry data into the Compound Discoverer 3.3 software (Thermo Fisher Scientific, USA) and analyzing it in conjunction with the BGI Metabolome Database (BMDB), mzCloud database, and ChemSpider online database, a comprehensive data matrix is generated. This matrix contains detailed information on metabolite peak areas and identification results. Subsequently, this data matrix undergoes further analysis and processing to refine and enhance the results. The specific parameters used in Compound Discoverer 3.3 include a parent ion mass deviation of less than 5 ppm, a fragment ion mass deviation of less than 10 ppm, and a retention time deviation of less than 0.2 min. For more information, the official website for Compound Discoverer can be accessed at: https://mycompounddiscoverer.com/. The data preprocessing steps included normalizing the data to obtain relative peak areas using Probabilistic Quotient Normalization (PQN), correcting batch effects with Quality control-based robust LOESS (QC-RLSC) signal correction and removing metabolites with a Coefficient of Variation larger than 30% on their relative peak area in QC Samples. Probabilistic Quotient Normalization (PQN) is a sample normalization method that enhances comparability between samples. It involves obtaining an overall reference vector by analyzing the ion intensity distribution in each sample and then analyzing the correction coefficient between the actual sample and the reference vector for actual sample correction. QC-RLSC, on the other hand, is an effective data correction method in the field of metabolomics. This method corrects experimental sample signals by locally fitting signal corrections based on QC sample information.

### Quality control

Principal component analysis (PCA) is an unsupervised pattern recognition method for statistical analysis of multidimensional data. This technique involves an orthogonal transformation that converts a set of potentially correlated variables into a new set of linearly uncorrelated variables, known as principal components (PC). In this study, PCA was applied to investigate how a few principal components can elucidate the internal structure among multiple variables while preserving the essential information from the original variables. The log transformation and Paretoscaling were mainly used to compute principal components. The PCA plot reflects the real distribution of samples and is mainly used to observe the separation trend between sample groups and whether there are abnormal samples, and to reflect the variability between groups and within groups from the original data. For quality control (QC) samples, the cohesion of these samples is indicative of instrument stability and data reproducibility. A more consolidated clustering of QC samples reflects better instrument stability and enhanced reproducibility in the collected data.

### Analysis of differential metabolites and functional pathways

Univariate analysis employed Fold Change (FC) and *p*-value to select differential metabolites. Fold Change (FC) was obtained through FC analysis, while the *p*-value was derived from the T-test. The *p*-value served as a metric to assess statistical significance between two analysis groups. The taxonomic and functional annotation of the identified metabolites provides valuable insights into the characteristics of distinct metabolites. The Human Metabolome Database (HMDB) contains comprehensive information, spanning chemical, molecular biology/biochemical, and clinical aspects of metabolites. It supports searches for metabolic pathways and spectral information. KEGG, or the Kyoto Encyclopedia of Genes and Genomes, serves as the central hub for numerous metabolic pathways and their interrelationships. Differential metabolites were assessed using MetaboAnalyst 5.0 (https://www.metaboanalyst.ca)^23^. The data were uploaded to the Kyoto Encyclopedia of Genes and Genomes (KEGG) web service (https://www.kegg.jp)^24^ and the Human Metabolome Database (HMDB) 4.0 (https://hmdb.ca)^25^ to obtain additional information for the identification of significantly altered pathways. The Small Molecule Pathway Database (SMPDB) (https://smpdb.ca/)^26^ was utilized for elucidating metabolic pathways through metabolite set enrichment analysis (MSEA)^27^ based on over-representation analysis (ORA). We employed the hypergeometric test, a widely accepted method, to identify pathways significantly enriched, with a threshold set at a *p*-value < 0.1, indicating significant enrichment. A smaller *p*-value indicates a more pronounced and significant enrichment in the identified pathways.

### Metabolite-metabolite interaction network

The metabolite-metabolite interaction network is a method designed to bring attention to potential functional relationships across a broad spectrum of annotated metabolites. In constructing this network, chemical-chemical associations were extracted from STITCH database^28^, with a focus on incorporating only the most reliable interactions. The basis for many of these associations in STITCH lies in co-mentions found in PubMed Abstracts, capturing connections involving reactions with similar chemical structures and parallel molecular activities. This approach ensures a robust and contextually relevant representation of metabolite interactions within the network.

### Statistical analysis

Statistical significance between the control sample and the treated groups was analyzed using unpaired, two-tailed Student’s t-test. All analyses were performed using the statistical program of GraphPad Prism software version 9 (San Diego, CA). Statistical significance levels were shown as * (P < 0.05), ** (P < 0.01), *** (P < 0.001), and **** (P < 0.0001).

## Results

### Cytotoxic effects of MH types on cancer cell lines

**Figure 1:**
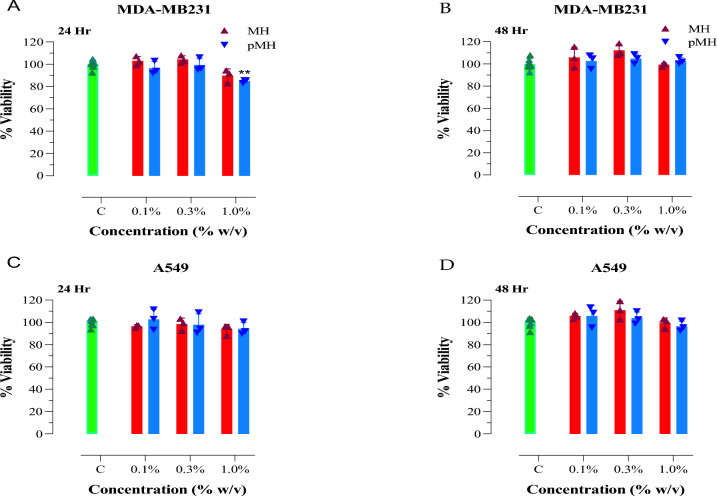
Minimal cytotoxic effect of raw MH or pMH against human breast and lung cancer cell lines. MDA-MB-231 breast cancer cells (**A**, **B**) and A549 lung cancer cells (**C**, **D**) were cultured alone or in the presence of MH or pMH at the indicated final concentrations for 24 h (**A**, **C**) or 48 h (**B**, **D**). The results are expressed as the percentage of viability (mean ± SD) of MH or pMH-treated cell cultures compared to untreated controls and are representative of two independent experiments. Asterisks denote statistically significant differences in viability between the experimental groups compared to control (**P < 0.01).

The cytotoxic effect of raw Manuka honey (MH) and powdered Manuka honey (pMH) against human breast and lung cancer cell lines was assessed after 24 or 48 h of culture (Fig. 1). At 24 h, no significant changes in cell viability were observed after treating MDA-MB-231 cells with up to 1% of raw MH (Fig. 1A). Similarly, treatment with pMH had only a small effect on vibility, causing a reduction of ~ 16% at the highest concentration (1% w/v) used (Fig. 1A). At 48 h, no inhibition in the viability of MDA-MB231 cells was observed after treatment with raw MH or pMH at up to 1% concentration (Fig. 1B). Similar findings were obtained when raw MH or pMH were tested on human A549 lung cancer cells, with treated cells showing essentially no reduction in viability compared to untreated cells (Fig. 1C,D).

**Figure 2:**
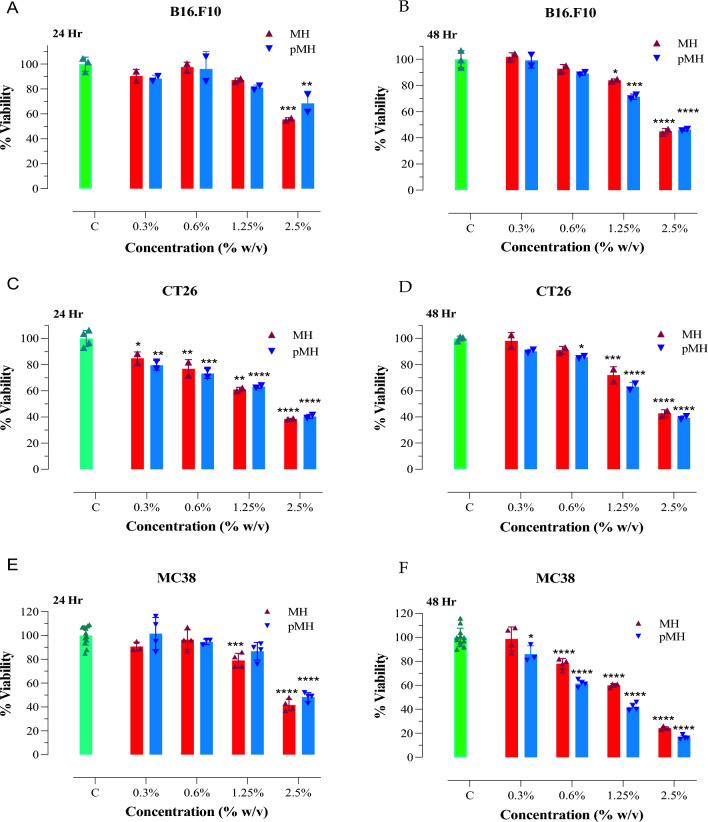
Comparable cytotoxic effect exhibited by MH and pMH on murine B16.F10 melanoma (**A**, **B**), CT26 colorectal (**C**, **D**) and MC38 colorectal adenocarcinoma (**E**, **F**) cell lines. Cells were cultured as described in Fig. 1 legend for 24 h (**A**, **C**, **E**) or 48 h (**B**, **D**, **F**). The results are expressed as the percentage of viability (mean ± SD) of MH or pMH-treated cell cultures compared to untreated controls and are representative of two independent experiments. Asterisks denote statistically significant differences in viability between the experimental groups compared to control (*P < 0.05; **P < 0.01; ***P < 0.001; ****P < 0.0001).

We next compared the effect of the two MH types on different murine cancer lines, including the B16.F10 melanoma, and CT26 and MC38 colorectal adenocarcinoma cells (Fig. 2). For these studies, we increased the range of MH concentrations to include 0.3%, 0.6%, 1.25%, and 2.5% (w/v). The findings showed that there was a dose-dependent reduction in viability in all 3 cancer cell lines after culture with either raw MH or pMH. The data also revealed a difference in suceptbility to MH between the cancer cell lines, with B16.F10 cells being more resistant (Fig. 2A,B) while CT26 (Fig. 2C,D) and MC38 (Fig. 2E,F) cells exhibiting a higher susceptibility to MH. The effect of MH and pMH on the proliferation of the three murine tumor cell lines was also assessed using the BrdU assay. The results showed a similar dose-dependent inhibition of cell proliferation by MH and pMH (Supplementary Fig. 1). Taken together, these findings demonstrate the differential susceptibility of the various human and mouse cancer lines to MH, with MC38 cells exhibiting the highest susceptibility among all of the cell lines tested and the MDA-MB-231 and A549 human cancer lines being least susceptible. Importantly, however, there was no evidence for any significant difference in the cytotoxic potential of raw MH compared to pMH on all of the tested cancer cell lines.

### Multivariate statistical analyses

**Figure 3:**
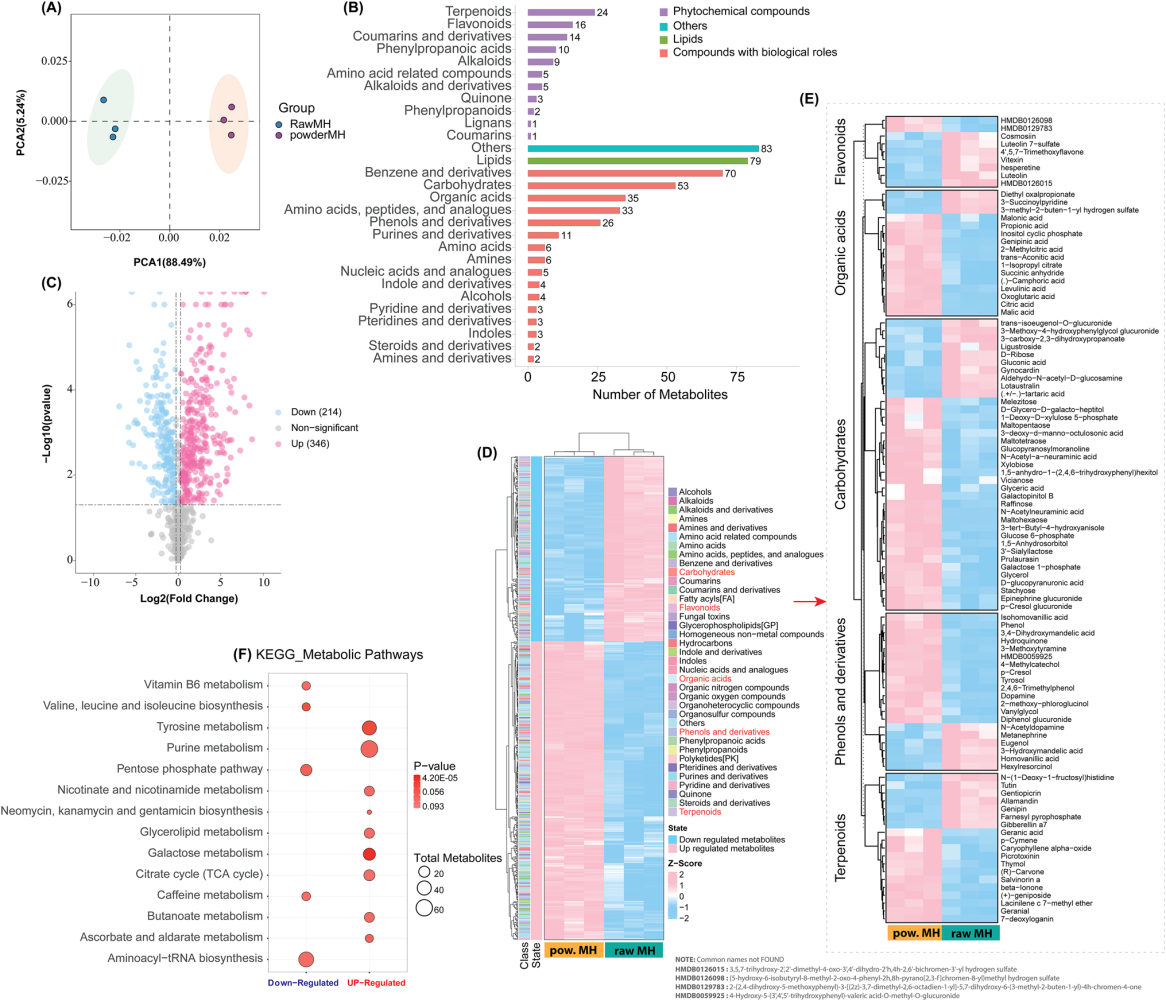
(**A**) The PCA scores plot reveals distinct clusters for powder MH and raw MH, demonstrating the robust repeatability of the data analysis. (**B**) The bar chart illustrates classification of metabolites. (**C**) Volcano plot illustrating differential metabolites. Each point represents a metabolite, with the x-axis indicating the logarithm of the quantitative difference multiples between pMH and rMH. The y-axis shows the -log10 p-value representing the significance of the difference. Light blue dots and pink dots denote down-regulated and up-regulated differentially expressed metabolites, respectively, while grey dots represent detected metabolites without significant differences. (**D**) The heatmap for metabolite clustering exhibits three biological replicates in the illustration. Powder MH is denoted as pMH1, pMH2, and pMH3, whereas raw MH is indicated as rMH1, rMH2, and rMH3. Various metabolites annotated in different classes, along with up- and down-regulated metabolites across all samples, are depicted by light blue and pink colors. (**E**) A sub- Heatmap depicting the abundance of key metabolites in Manuka honey, including carbohydrates, flavonoids, organic acids, phenols and derivatives, and terpenoids. This comprehensive visualization highlights the diverse bioactive compounds that contribute to Manuka honey's energy-providing properties, antioxidant and anti-inflammatory effects, acidity regulation, antimicrobial activity, and aromatic profile. (**F**) Pathway enrichment analyses of upregulated or downregulated metabolites in pMH group compared with the raw-MH group. The size of each dot represents the number of metabolites involved in the current KEGG metabolic pathway. The color of the dot indicates the p-value from the hypergeometric test, with darker colors indicating greater significance and lighter colors indicating lower significance.

We performed untargeted metabolomics analysis of raw MH and pMH using UHPLC-Q-TOF–MS to determine their characteristics and uncover any potential differential metabolomics content. Multivariate statistical analyses, including principal component analysis (PCA), were performed to assess the reliability of the obtained sample data. The two principal components described a total variance of 93.73% (PCA1 88.48% and PCA2 5.24%). The score plot visually illustrates the similarity among triplicate samples of each MH types (pMH and raw MH) and the clear distinctions between the two types of honey samples, thereby validating the quality of the metabolomics data and highlighting a relatively high level of reproducibility in this assessment (Fig. 3A).

### Differential metabolites and metabolic pathway analysis

The statistical representation of metabolite numbers and identification post-data preprocessing revealed the detection of 2440 compounds, of which only 833 were identified, and listed in Supplementary File 1. The distribution of metabolites with identification information is presented based on their final class. It is noteworthy that not every metabolite possesses final class information; for lipids, molecular subclass information was utilized instead. The analysis highlighted the identification of 90 phytochemical compounds, with terpenoids, flavonoids, coumarins and derivatives, and phenylpropanoic acids being the most abundant, constituting of 24, 16, 14, and 10 compounds, respectively (Fig. 3B). Additionally, 79 lipid metabolites were identified. Among compounds with biological roles, there were 266, including benzene and derivatives, carbohydrates, organic acids, amino acids and peptides, phenols, and other derivatives (Fig. 3B).

The differential metabolites are visually represented through the volcano plot, showing significant levels of metabolites in raw MH compared to pMH. The threshold values were set as abs|log2FC > 0.25|, and p value < 0.05 in order to capture all metabolites with statistically significant differences between raw MH and pMH, ensuring no meaningful changes were overlooked while maintaining statistical rigor. As shown in Fig. 3C, a total of 560 significant metabolites were identified, with 346 upregulated (pink circle-shadowed) and 214 downregulated (light blue circle-shadowed) metabolites, revealing significant differential changes between the MH types. Additionally, we conducted hierarchical clustering utilizing the Euclidean distance method to check the expression patterns of each differential metabolite across samples. In this representation, each row corresponds to a metabolite, each column corresponds to a sample, and color signifies the expression level. Substantial differences in metabolite content between the two MH types are clearly evident (Fig. 3D). By focusing on the top 30 metabolites up or down in abundance, we identified 5 major classes of metabolites with differential representation in the two types of MH, namely carbohydrates, flavonoids, phenols and derivatives, organic acids, and terpenoids. Hierarchical clustering of the extent of the abundance of individual metabolites within these 5 classes is shown in Fig. 3E.

In addition, the pathway enrichment analyses showed that the pentose phosphate pathway, valine, leucine and isoleucine biosynthesis, aminoacyl-tRNA biosynthesis, vitamin B6 metabolism, and caffeine metabolism were significantly decreased in pMH. By contrast, galactose metabolism, tyrosine metabolism, ascorbate and aldarate metabolism, purine metabolism, neomycin, kanamycin and gentamicin biosynthesis, butanoate metabolism, nicotinate and nicotinamide metabolism, glycerolipid metabolism and citrate cycle (TCA cycle) were increased in pMH compared with the raw MH group (Fig. 3F).

### Metabolic interaction networks (MIN)

**Figure 4:**
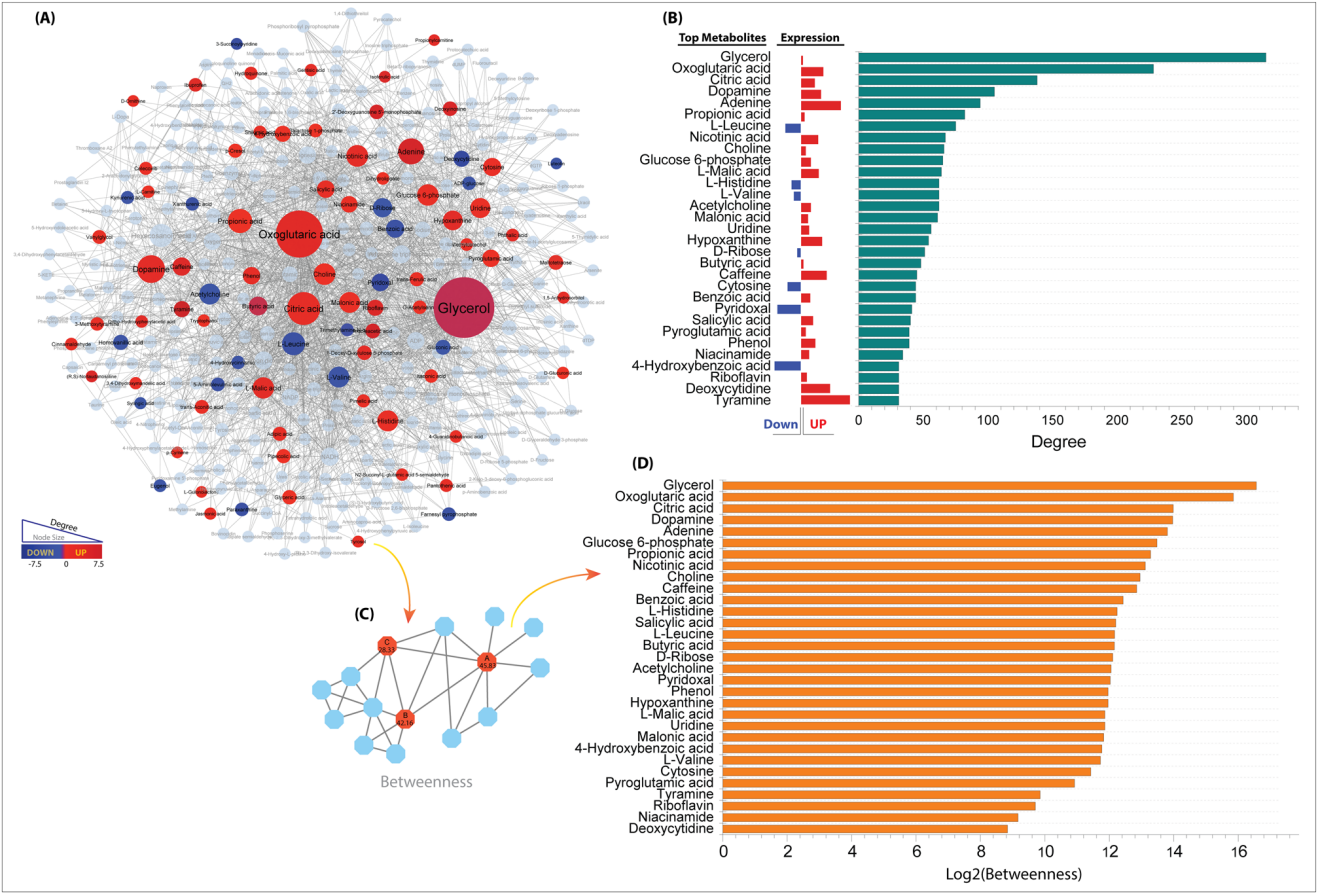
(**A**) Representation of metabolite-metabolite interactions: Metabolites are depicted as nodes (circles) in the network. Node colors denote expression patterns; for instance, red and blue signify upregulated and downregulated metabolites, respectively, while other interacting metabolites are presented in light grey. The size of each node reflects its node degree, indicating the number of links a node has to other nodes. (**B**) Bar graph illustrating the degrees of the top 30 metabolites in the network, with their expression patterns depicted in red for upregulated and blue for downregulated metabolites. (**C**). Schematic illustration depicting the importance of betweenness centrality, highlighting that node A, B, C possesses the highest betweenness values, exerting a significant impact on the global network. (**D**) Bar graph visualizing the betweenness centrality of the top 30 metabolites in MMI network.

We conducted an analysis of the Metabolic Interaction Network (MIN) using differential metabolites, establishing links between them if they are connected by at least more than two other metabolites or reactions. Upon scrutinizing the network's topological properties, we observed that the distribution of metabolite connectivity follows a scale-free pattern, indicative of a power-law distribution. In simpler terms, a few metabolites within the network exhibit significantly higher connectivity, while the majority have relatively few connections (Fig. 4A). Consequently, we identified the top 30 key metabolites with a degree > 30 and high betweenness centrality that act as important bridges or intermediaries within the network (Fig. 4B–D). This underscores their crucial roles in regulating metabolic pathways, implying that alterations in these metabolites could potentially lead to significant changes in the bioactivity of the MH samples.

## Discussion

The mechanism by which MH exerts its cancer-healing properties is multifaceted. In addition to its direct anti-proliferative capacity through the activation of the intrinsic and extrinsic apoptotic pathways^19,20^, MH regulates immune responses by ameliorating hematological and serological restrictions and activates the intrinsic apoptotic pathway through the modulation of pro-apoptotic protein expression^5^. The effective inhibitory role of MH at low concentrations on the growth of several cancer cell types, such as melanoma, colorectal cancer, and breast adenocarcinoma, has been reported^21^. Furthermore, in an implantable melanoma preclinical model, systemic management of MH improved the anti-tumor paclitaxel activity and enhanced the overall survival of the host. The anti-tumor activity of several honey types on cancer cells has also been investigated^29^. The pro-apoptotic and anti-proliferative features of honey on cancer cells are assumed to be chiefly attributed to its phenolic compounds content, comprising quercetin, luteolin, chrysin, and caffeic acid esters^30^. It was also established that honey had the ability to induce caspase-mediated apoptosis in diverse cancer cell lines, for instance breast, melanoma, prostate, liver, cervical, and renal cancers^31,32^. Nevertheless, the mechanism of the initial upstream target in cancer cells influenced by honey treatment needs further clarification.

We previously reported that the IC_50_ value (the concentration that results in 50% inhibition in cell viability) of MH (UMF 10+) against CT26 colon cancer cells was ~ 20 and 10 mg/mL, at 24 and 72 h of culture, respectively^21^. Despite the fact that we used UMF 20 + MH in this study, the IC_50_ values against the same cell line were similar, calculated to be 19 mg/ml and 21.5 mg/ml at 24h and 48h, respectively. The equivalent values for pMH were 19.6 mg/ml and 19.8 mg/ml, respectively. Comparing the values for MH with other honey types, the estimated IC_50_ values of Gelam honey, Nenas honey, and Indian commercial honey were 39–80 mg/mL, 85.5 mg/mL, and 35–40 mg/mL, on human colon cancer HT-29, HCT-15, and HCT-116 cells, respectively, at 24 h^32,33^. These levels were higher compared to MH in the present work. The observed variations could be mainly attributed to several factors, such as the composition of honey, precisely the diverse varieties of phenolic acids and flavonoids, known as chemo-preventive mediators^34^. Based on different literature, phenolic compounds, for instance, quercetin, gallic acid, luteolin, kaempferol, and caffeic acid that are also detected in MH, have fundamental roles in the suppression of cancer cell proliferation^21,34^. In a previous study, we evaluated the early targets of MH and its modulatory properties on the proliferation, invasiveness, and angiogenic potential using two human breast cancer cell lines, the triple-negative MDA-MB-231 cells, and estrogen receptor-positive MCF-7 cells, and the non-neoplastic breast epithelial MCF-10A cell line. The study revealed that while exposure to MH at levels of 0.3–1.25% had no to minimal effect on cell proliferation, significant dose-dependent inhibition of cell migration, colony formation and invasion was observed^20^. In the same study, we reported that at concentrations higher than 2.5% (or 25 mg/ml), MH was found to be toxic to the proliferation of all three cancer cell lines^20^.

Honey is mainly composed of sugars, water, and other constituents, for instance, proteins, minerals, vitamins, organic acids, volatile compounds, and phenolic compounds, which can be dissolved and stabilized in an aqueous phase; accordingly, honey can also be dissolved in the extraction solvent methanol/water^35^. It was previously reported that adding formic acid to the extraction solvents resulted in higher responses and sharp peaks in chromatographic separation as it provides the protons to form [M + H]+ ions for analysis under the positive ionization mode^36^. Another study also revealed that formic acid at low concentrations could improve the stability of the extracted metabolites^37^. Therefore, a mixture of methanol/water comprising 0.1% formic acid was selected for the extraction of honey metabolites.

In this study, UPLC-Q-TOF-MS -based untargeted metabolomics analysis was applied to identify the metabolite characteristics profile of raw MH and pMH. The indicative retention times, mass-to-charge ratios of precursor ions in both positive and negative ion modes, and MS–MS fragment ions, along with the intensities of the identified ions, serve as distinct electro-spray ionization mass spectrometry (ESI–MS) fingerprints for the two MH types. Nevertheless, the classification of all analytical ions proved challenging, as the mass-to-charge values of precursor ions could not be reconciled with MS–MS fragment ions within existing databases. Sun et al.^38^ analyzed mature and immature honey using LC–MS metabolomics analysis and determined the potential fatty acids marker by using GC–MS. It was shown that a total of nine discriminating metabolites, including bee-originated and plant-originated components, could be supportive to differentiate these two types of honey through metabolomics data analysis.

In our study, a thorough analysis confirmed 833 distinct metabolites. Among these, a total of 560 significant metabolites were identified, with 346 showing upregulation and 214 showing downregulation. These findings highlight substantial differential changes between the two types of MH. These metabolites encompass various classes, including carbohydrates, flavonoids, phenols and derivatives, organic acids, and terpenoids. It is interesting to note that while raw MH was enriched in carbohydrates and flavonoids, pMH had increased abundance of phenols and derivatives, organic acids, and terpenoids. All of these metabolite classes contain bioactive compounds that possess anti-cancer properties, either directly or indirectly. For example, carbohydrates, which were the most abundant in our dataset, contain many non-digestible carbohydrates (e.g. Melezitose, Raffinose, Stachyose and Xylobiose) which have shown to effect changes in the gut micrbiota that confer health benefits to the host. These benefits are associated with increased numbers of beneficial microbes like bifidobacteria and lactobacilli in the gut, and increased production of metabolites such as short-chain fatty acids (SCFA) by gut microbes^39^. The beneficial properties of flavonoid compounds as anticancer agents are well studied and have been extensively reviewed^5,40^. Many phenols and phenolic acids have anti-inflammatory, anti-metastatic, and anticancer properties^41^. Additionally, honey polyphenols can improve intestinal inflammation and oxidative stress resistance by modulating gut microbiota^42^. It is of interest to note that organic acids, including Malonic acid, Propionic acid, Genipinic acid, 2-Methylcitric acid, trans-Aconitic acid, Camphoric acid, Levulinic acid, Oxoglutaric acid, Citric acid, and Malic acid, are more abundant in pMH compared to raw honey. These organic acids help in stabilizing the composition of MH and play a role in its antimicrobial properties^43^. Terpenoids represent the largest and most diverse group of naturally occurring phytoconstituents, possessing a range of pharmacological activities, including anticancer effects^44^. Coumarin derivatives, for instance, have been shown to induce apoptosis by up-regulating caspase 3 and caspase 9 expression, while also exhibiting antiproliferative and anti-metastatic effects through PAK1 and PAK2-mediated signaling^45^.

Furthermore, the metabolic pathway analysis showed that the identified metabolites were mostly involved in amino acid metabolism, xenobiotics biodegradation and metabolism, carbohydrate metabolism, terpenoids polyketides metabolism, lipids metabolism, nucleotide metabolism, and energy metabolism. Among the most significant differentially expressed metabolic pathways observed in pMH were tyrosine and purine metabolism which were upregulated and the pentose phosphate pathway and aminoacyl-tRNA biosynthesis which were downregulated. These alterations in metabolite contents between the two types of MH could induce preferential effects on cancer cells. It is important to emphasize that all of these metabolic pathways are intricately involved in the regulation of cancer growth^46–49^.

## Conclusions

The multifaceted healing properties of Manuka honey (MH) in combating cancer are evident through its regulation of immune responses, amelioration of hematological and serological restrictions, and activation of the intrinsic apoptotic pathway. The inhibitory impact of MH on various cancer cell types, including melanoma, colorectal cancer, and breast adenocarcinoma, underscores its potential as a therapeutic agent. Moreover, the observed variations in the induced IC50 values of MH compared to other honey types emphasize the significance of honey composition, particularly the diverse varieties of phenolic acids and flavonoids, as crucial chemopreventive mediators. The pro-apoptotic and anti-proliferative features of honey, attributed to phenolic compounds like quercetin, gallic acid, luteolin, kaempferol, and caffeic acid, contribute to its efficacy in suppressing cancer cell proliferation across diverse cell lines. Additionally, our comprehensive metabolomics analysis of MH and its comparison with other honey types shed light on the intricate metabolic pathways involved. The identification of 833 differential metabolites, encompassing polyphenols, carbohydrates, lipids, and other compounds, elucidates the rich biochemical profile of MH. A deeper understanding of the metabolite characteristics, including involvement in amino acid metabolism, xenobiotics biodegradation, carbohydrate metabolism, terpenoids polyketides metabolism, lipids metabolism, nucleotide metabolism, and energetic metabolism, paves the way for future investigations into the molecular mechanisms underlying MH's anticancer and immunomodulatory activities. As chemometric approaches continue to refine our ability to differentiate honey samples from diverse entomological origins, our study contributes to the growing body of knowledge surrounding the therapeutic potential of honey, particularly MH, in the realm of cancer research.

### Supplementary Information

Supplementary Figure 1.

Supplementary Information.

## Data Availability

The datasets used and/or analysed during the current study available from the corresponding author on reasonable request.
