# Supplementary material for: Differential in vitro cytotoxic effects and metabolomic insights into raw and powdered Manuka honey through UPLC-Q-TOF-MS
Source: Sci Rep. 2024 Jul 30;14:17551. doi: 10.1038/s41598-024-68387-7 (PMC11289323; doi:10.1038/s41598-024-68387-7)
Supplement: Supplementary file 1 — Supplementary Figure 1. [file 41598_2024_68387_MOESM1_ESM.docx]

**Differential In Vitro Cytotoxic Effects and Metabolomic Insights into Raw and Powdered Manuka Honey through UPLC-Q-TOF-MS**

**Ienas Idriss^a^, Abdelmoneim H. Ali^b^, Aftab Alam^a^, Maria Fernandez-Cabezudo^c,e^,** **Mutamed Ayyash^d,e^ Basel K. al-Ramadi^a,e,f^**

^a^ Department of Medical Microbiology and Immunology, College of Medicine and Health Sciences, United Arab Emirates University (UAEU), Al Ain, UAE

^b^ Department of Food Science, Faculty of Agriculture, Zagazig University, 44511 Zagazig, Egypt

^c^ Department of Biochemistry & Molecular Biology, College of Medicine and Health Sciences, United Arab Emirates University (UAEU), Al Ain, UAE

^d^ Department of Food Science, College of Agriculture and Veterinary Medicine, United Arab Emirates University (UAEU), Al Ain, P.O. Box 15551, UAE

^e^ Zayed Center for Health Sciences, United Arab Emirates University (UAEU), Al Ain, UAE

^f^ ASPIRE Precision Medicine Research Institute Abu Dhabi, United Arab Emirates University, Al Ain, United Arab Emirates.


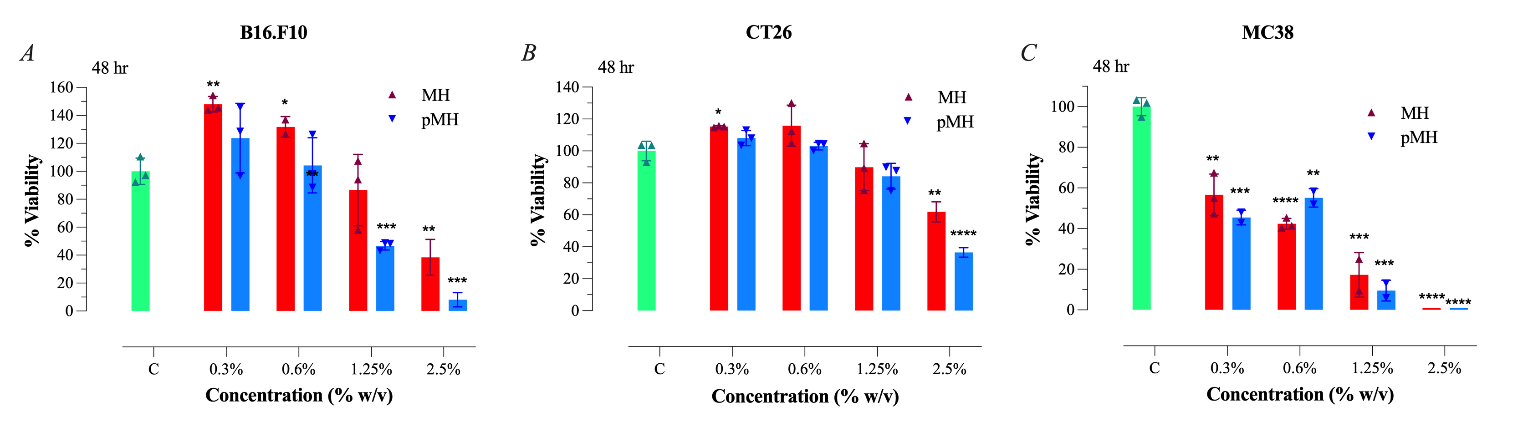


**Supplementary Figure 1.** Effect MH and pMH on the proliferation of murine B16.F10 melanoma (A), CT26 colorectal (B) and MC38 colorectal adenocarcinoma (C) cell lines as measured by BrdU incorporation. Cells were cultured as described in M&M for 48 h. The results are expressed as the percentage of viability (mean ± SD) of MH or pMH-treated cell cultures compared to untreated controls and are representative of two independent experiments. Asterisks denote statistically significant differences in viability between the experimental groups compared to control (**P* < 0.05; ***P* < 0.01; ****P* < 0.001; *****P* < 0.0001).
